# Association Between Prescription Pain Medication Use and Cardiovascular Risk Factors in US Adults: A Cross-Sectional Study Using the National Health and Nutrition Examination Survey (2017-2020)

**DOI:** 10.7759/cureus.108951

**Published:** 2026-05-16

**Authors:** Moses C Odoeke, Osatohanmwen Irorere, Oluwatobiloba Omotunde, Kimberly Osias, Marie R Bonhomme, Emeka Okwuokei, Ikenna C Madubuike, Emmanuel C Nwaokobia, Akintunde C Akinboboye

**Affiliations:** 1 Internal Medicine, University of Toledo, Toledo, USA; 2 Psychiatry, Neurology, and Internal Medicine, Caribbean Medical University, Willemstad, CUW; 3 Internal Medicine, American International School of Medicine, Atlanta, USA; 4 Family Medicine, Université Notre Dame d'Haïti, Port-au-Prince, HTI; 5 Medicine, Faculty of Medicine and Pharmacy, Université Notre Dame d'Haïti, Port-au-Prince, HTI; 6 Internal Medicine, Hywel Dda University Health Board, Aberystwyth, GBR; 7 Operations, Favored Healthcare Services, Buford, USA; 8 Carey Business School, Johns Hopkins University, Lawrenceville, USA; 9 Family Medicine, 168 Medical Group, Weston-super-Mare, GBR; 10 Emergency, University of Medical Sciences Teaching Hospital, Ondo, NGA

**Keywords:** cardiovascular risk, nhanes, opioid use, pain medication, risk factors, united states

## Abstract

Background

Cardiovascular disease (CVD) remains a leading cause of morbidity and mortality in the United States, with risk driven by the clustering of modifiable factors such as hypertension, diabetes, obesity, dyslipidemia, and smoking. Prescription pain medication use is common among adults with chronic conditions, yet its relationship with cardiovascular risk burden is not well-characterized.

Objective

The objective of this study is to evaluate the association between prescription pain medication use and cardiovascular risk burden among adults in the United States.

Methods

This cross-sectional study used data from the National Health and Nutrition Examination Survey (NHANES) 2017 to March 2020 prepandemic cycle. Adults aged 18 years and older were included. The exposure was prescription pain medication use. The outcome was a composite cardiovascular risk burden score categorized into low, moderate, and high risk. Survey-weighted logistic regression was used to examine the association with high cardiovascular risk, adjusting for demographic and socioeconomic factors. The final analytic sample included 6,662 participants, corresponding to a weighted population of 195,893,244 US adults.

Results

Prescription pain medication use was associated with higher odds of high cardiovascular risk factor burden (adjusted odds ratio, 1.54; 95% confidence interval (CI), 1.15-2.07; p = 0.005). Increasing age was associated with higher odds (adjusted odds ratio, 1.02; 95% confidence interval, 1.01-1.03; p = 0.003). A higher income-to-poverty ratio was associated with lower odds (adjusted odds ratio, 0.92; 95% confidence interval, 0.85-0.99; p = 0.029). Physical activity was associated with lower odds (adjusted odds ratio, 0.53; 95% confidence interval, 0.39-0.74; p < 0.001).

Conclusion

Prescription pain medication use is associated with a higher cardiovascular risk factor burden among US adults. These findings support the need for integrated cardiovascular risk assessment in individuals receiving pain medications.

## Introduction

Cardiovascular disease (CVD) remains the leading cause of morbidity and mortality in the United States, with annual direct and indirect costs estimated at $407.3 billion [1]. Despite some significant progress in the prevention and treatment, the cardiovascular risk factor burden among adults in the United States continues to increase with the presence of hypertension, dyslipidemia, obesity, and diabetes mellitus [2,3]. All these risk factors are not only lifestyle and genetically predisposed but also medication-dependent, becoming a major yet under-researched risk factor that influences the outcomes of cardiovascular health [4,5].

The clinical use of prescription pain medications to treat acute and chronic pain conditions is common in clinical practice [6]. These medications include opioids, nonsteroidal anti-inflammatory drugs (NSAIDs), and certain adjuvant pain relievers such as anticonvulsants and antidepressants [7]. The use of prescription pain medications and, in particular, opioids has taken up a huge portion of the prescription drug use in the United States over the past two decades due to concern over their safety profile in the long term, in addition to the dependency and misuse of these drugs [8,9]. Although these drugs are essential in enhancing the quality of life and functional status, there has been a growing body of evidence that some classes could have undesirable effects on cardiovascular health [10].

Nonsteroidal anti-inflammatory drugs have always been associated with increased risks of increased blood pressure, fluid retention, and adverse cardiovascular effects, including myocardial infarction and stroke [11]. Similarly, metabolism, decreased physical activity, and possible endocrine distortions have been associated with opioid consumption, which could indirectly lead to cardiovascular risk [12]. Importantly, individuals who experience chronic pain that does not go away are more likely to have comorbidities such as obesity, sedentary lifestyle, and psychological stress, which normally exacerbates their cardiovascular risk profile further [13]. Notwithstanding these apprehensions, the manner of the interwoven nature of prescription pain drug consumption with the overall burden of cardiovascular risk factors in the population setting is not properly defined [14,15].

The opioid crisis poses a public health problem contributed by opioid misuse, which is characterized by the use of opioids in another way or amount than what is prescribed, using another person's prescription, or even using opioids to experience euphoria [16]. However, this may inform clinical decision-making, risk stratification, and targeted interventions regarding whether people taking prescription pain medications are more prone to having poor cardiovascular risk profiles [17]. Major adverse cardiovascular events are usually defined as acute myocardial infarction (AMI), stroke, heart failure, and cardiovascular mortality [18]. Furthermore, these understandings can be crucial in the process of weighing the advantages of pain management against the possible long-term health outcomes, in particular in the framework of chronic disease prevention [19].

The study employs National Health and Nutrition Examination Survey (NHANES) data. The NHANES includes data on the use of prescription medications, clinical measures, and laboratory results in detail, which makes it possible to thoroughly assess cardiovascular risk factors and prescription medication exposure [20]. The objective of the study is to evaluate whether prescription pain medication use is associated with a higher burden of cardiovascular risk factors among adults in the United States. While prior studies have examined individual cardiovascular outcomes, less is known about the overall clustering of risk factors at the population level. This study adds to the existing literature by using nationally representative NHANES data to evaluate the clustering of cardiovascular risk factors through a composite risk burden measure. While prior studies have focused on individual cardiovascular outcomes or specific medication classes, this approach provides a broader population-level perspective on the relationship between prescription pain medication use and overall cardiometabolic risk.

## Materials and methods

Study design and data source

This study used a cross-sectional design based on data from the National Health and Nutrition Examination Survey conducted during the 2017 to March 2020 prepandemic cycle [21]. The NHANES is a nationally representative survey of the noninstitutionalized US population that combines interviews, physical examinations, and laboratory assessments. Data are collected using a complex multistage probability sampling design that incorporates stratification, clustering, and sampling weights to ensure national representativeness [21]. Publicly available datasets from demographic, examination, laboratory, and questionnaire components were merged using the unique participant identifier.

Study population

The study population was restricted to adults aged 18 years and older. After this restriction, the initial analytic sample included 15,560 participants with available demographic data. The participants were included if they had information on prescription medication use and at least partial data on cardiovascular risk factors. A complete case analytic dataset was constructed by restricting the sample to participants with non-missing data on the exposure, outcome, and selected covariates. This resulted in a final unweighted analytic sample of 6,662 participants. All analyses incorporated survey sampling weights, and the weighted population size represented 195,893,244 US adults.

Variables and measures

The primary exposure was prescription pain medication use, defined as a binary variable indicating any reported use of commonly prescribed analgesic medications. This variable was derived from reported drug names and included opioid medications such as morphine, oxycodone, hydrocodone, fentanyl, codeine, and tramadol, as well as selected non-opioid analgesics including ibuprofen, naproxen, gabapentin, and pregabalin, consistent with prior studies examining prescription medication use in the NHANES [6,8]. Participants reporting the use of any of these medications were classified as exposed.

The primary outcome was a composite cardiovascular risk burden measure. Five established cardiovascular risk factors were defined using clinical and self-reported data. Hypertension was defined as a mean systolic blood pressure of at least 130 mmHg, a mean diastolic blood pressure of at least 80 mmHg, or self-reported physician diagnosis [4]. Diabetes was defined as glycated hemoglobin of at least 6.5%, fasting plasma glucose of at least 126 mg/dL, or self-reported diagnosis [5]. Dyslipidemia was defined using a simplified approach as total cholesterol of at least 200 mg/dL or low high-density lipoprotein cholesterol, defined as less than 40 mg/dL in men or less than 50 mg/dL in women [2,3]. Obesity was defined as a body mass index of at least 30 kg/m^2^ [3]. Current smoking was defined as having smoked at least 100 cigarettes in a lifetime and currently smoking every day or some days, consistent with standard epidemiologic definitions used in the NHANES [21]. A composite cardiovascular risk burden score was calculated by summing the presence of these five risk factors, yielding a range from 0 to 5. This approach reflects cumulative cardiometabolic risk and is consistent with prior epidemiologic studies that assess the clustering of risk factors rather than individual conditions [2,3]. The score was categorized into low risk for 0-1 factor, moderate risk for 2-3 factors, and high risk for 4 or more factors. For regression analysis, a binary outcome variable indicating high cardiovascular risk was created.

Covariates included age, sex, race or ethnicity, education level, income-to-poverty ratio, health insurance status, and physical activity. Race or ethnicity and education were modeled as categorical variables, with non-Hispanic White and college education or higher specified as reference groups. Physical activity was defined as engagement in either moderate or vigorous activity based on questionnaire responses.

Missing data

Missing data were assessed for all variables included in the analysis. The proportion of missing data was 14.15% for the income-to-poverty ratio, 40.76% for education, 41.77% for hypertension, 5.12% for diabetes, 15.57% for obesity, 37.74% for smoking status, 37.72% for physical activity, and 0.24% for health insurance status. The composite cardiovascular risk score had 9.87% missingness after applying a requirement of at least three non-missing components.

To ensure consistency across analyses and avoid variation in analytic sample size between models, a complete case approach was implemented. A binary indicator identifying the participants with non-missing values for the exposure, outcome, and all covariates was generated, and the participants with missing data were excluded from the analytic dataset. This resulted in a final analytic sample of 6,662 participants. Although this approach reduced the sample size, the remaining dataset retained sufficient statistical power and national representativeness to address the study objective. The large initial sample size and the use of survey weights supported stable estimation. The potential for selection bias due to nonrandom missingness was acknowledged in the limitations and considered during interpretation.

Statistical analysis

All analyses accounted for the complex NHANES survey design using sampling weights, strata, and primary sampling units. Descriptive statistics were used to summarize participant characteristics. Continuous variables were expressed as means with standard deviations, and categorical variables were presented as frequencies and column percentages. Group comparisons by the outcome variable were conducted using survey-adjusted t tests for continuous variables and survey-adjusted chi-square tests for categorical variables. The primary analysis used survey-weighted logistic regression to examine the association between prescription pain medication use and high cardiovascular risk. The adjusted model included age, sex, race or ethnicity, education level, income-to-poverty ratio, health insurance status, and physical activity as covariates selected a priori based on their established association with cardiovascular risk. Variables used to construct the CVD composite outcome were not included in regression models to avoid overadjustment and model instability. Multicollinearity was assessed using variance inflation factors (VIF), and VIF values ranged from 1.02 to 1.35, with a mean VIF of 1.19, indicating no evidence of problematic multicollinearity. Results were reported as odds ratios with 95% confidence intervals (CI), and statistical significance was defined as a two-sided p-value of less than 0.05. All statistical analyses were carried out using Stata version 18 (StataCorp LLC, College Station, TX) [22].

Ethical considerations

NHANES data are publicly available and deidentified, and all participants provided informed consent at the time of data collection. The use of this dataset does not constitute human subject research requiring institutional review board approval.

## Results

Table 1 presents the baseline characteristics of the study population stratified by prescription pain medication use.

**Table 1:** Baseline characteristics of the participants by prescription pain medication use Values are weighted counts and column percentages for categorical variables and weighted mean and standard deviation (SD) for continuous variables. GED refers to general educational development. Group comparisons were conducted using survey-adjusted t tests for continuous variables and survey-adjusted chi-square tests with design-based F statistics for categorical variables. The asterisk (*) indicates statistical significance at p<0.05; "-" indicates intentionally left blank. The table was generated by the authors using Stata version 18 [22] CVD: cardiovascular disease

Characteristic	No Pain Medication (N = 176,757,720)	Pain Medication Use (N = 19,135,524)	Test Statistic	P-value
Age (years), mean ± SD	47.78 ± 17.09	55.54 ± 16.81	t = -7.34	<0.001*
Income-to-poverty ratio, mean ± SD	3.19 ± 1.62	2.64 ± 1.68	t = 6.17	<0.001*
Gender, n (%)	-	-	F(1,25) = 11.55	0.002*
Male	86,902,679 (49%)	7,533,990 (39%)	-	-
Female	89,855,041 (51%)	11,601,534 (61%)	-	-
Race/ethnicity, n (%)	-	-	F(3.48,86.93) = 10.03	<0.001*
Mexican American	13,763,238 (8%)	860,676 (4%)	-	-
Other Hispanic	12,358,447 (7%)	1,204,911 (6%)	-	-
Non-Hispanic White	115,138,599 (65%)	13,235,960 (69%)	-	-
Non-Hispanic Black	19,186,460 (11%)	1,967,237 (10%)	-	-
Non-Hispanic Asian	9,801,499 (6%)	292,259 (2%)	-	-
Other race	6,509,477 (4%)	1,574,481 (8%)	-	-
Education level, n (%)	-	-	F(1.79,44.80) = 8.20	0.001*
Less than high school	17,087,774 (10%)	2,195,972 (11%)	-	-
High school/GED	46,488,743 (26%)	6,524,488 (34%)	-	-
College or above	113,181,203 (64%)	10,415,064 (54%)	-	-
Health insurance, n (%)	-	-	F(1,25) = 20.37	<0.001*
Uninsured	22,951,855 (13%)	914,603 (5%)	-	-
Insured	153,805,865 (87%)	18,220,921 (95%)	-	-
Smoking status, n (%)	-	-	F(1,25) = 19.99	<0.001*
Nonsmoker	148,204,597 (84%)	14,963,260 (78%)	-	-
Smoker	28,553,123 (16%)	4,172,264 (22%)	-	-
Physical activity, n (%)	-	-	F(1,25) = 30.20	<0.001*
Not active	75,143,837 (43%)	12,182,931 (64%)	-	-
Active	101,613,883 (57%)	6,952,593 (36%)	-	-
Obesity, n (%)	-	-	F(1,25) = 13.17	0.001*
No	103,596,560 (59%)	8,348,765 (44%)	-	-
Yes	73,161,160 (41%)	10,786,759 (56%)	-	-
Diabetes, n (%)	-	-	F(1,25) = 36.39	<0.001*
No	156,369,196 (88%)	14,614,457 (76%)	-	-
Yes	20,388,524 (12%)	4,521,067 (24%)	-	-
Hypertension, n (%)	-	-	F(1,25) = 57.49	<0.001*
No	87,365,637 (49%)	5,408,860 (28%)	-	-
Yes	89,392,083 (51%)	13,726,664 (72%)	-	-
Dyslipidemia, n (%)	-	-	F(1,25) = 1.67	0.209
No	83,964,864 (48%)	8,348,270 (44%)	-	-
Yes	92,792,856 (52%)	10,787,254 (56%)	-	-
CVD risk category, n (%)	-	-	F(1.72,43.10) = 30.74	<0.001*
Low	77,018,258 (44%)	4,615,713 (24%)	-	-
Moderate	88,719,366 (50%)	11,964,715 (63%)	-	-
High	11,020,096 (6%)	2,555,096 (13%)	-	-

The findings from the table above indicate clear differences between the participants who reported prescription pain medication use and those who did not. Participants using pain medication were older, with a mean age of 55.54 (16.81) years compared to 47.78 (17.09) years among non-users, and had a lower income-to-poverty ratio of 2.64 (1.68) versus 3.19 (1.62), both statistically significant (p < 0.001). A higher proportion of women was observed among medication users, 11,601,534 (61%) compared to 89,855,041 (51%) in non-users. Differences in race and ethnicity were significant, with a higher proportion of non-Hispanic White participants among users, 13,235,960 (69%), while Mexican American and non-Hispanic Asian groups were less represented. Educational attainment differed across groups, with fewer individuals with a college education among users, 10,415,064 (54%) compared to 113,181,203 (64%) in non-users.

Health insurance coverage was more common among medication users, 18,220,921 (95%) compared to 153,805,865 (87%) in non-users. Current smoking was more frequent among users, 4,172,264 (22%) compared to 28,553,123 (16%) in non-users. Physical inactivity was also more common among users, 12,182,931 (64%) compared to 75,143,837 (43%) in non-users. Clinical risk factors were consistently more prevalent among those using pain medication. Obesity was present in 10,786,759 (56%) of users compared to 73,161,160(41%) of non-users. Diabetes was observed in 4,521,067 (24%) of users versus 20,388,524 (12%) of non-users. Hypertension was more frequent among users, 13,726,664 (72%) compared to 89,392,083 (51%) in non-users. Dyslipidemia did not differ significantly between groups.

The distribution of cardiovascular risk categories showed a higher proportion of individuals in the high-risk group among medication users, 2,555,096 (13%) compared to 11,020,096 (6%) in non-users, while low risk was less common among users. These differences were statistically significant (p < 0.001).

Table 2 presents the results of the multivariable logistic regression analysis examining factors associated with high cardiovascular risk.

**Table 2:** Multivariable logistic regression for high cardiovascular risk Results are presented as adjusted odds ratios with 95% confidence intervals (CI). Models were estimated using survey-weighted logistic regression accounting for strata, clusters, and sampling weights. Reference categories were male for sex, college education or higher for education, and non-Hispanic White for race or ethnicity. GED refers to general educational development; "-" indicates intentionally left blank. The asterisk (*) denotes statistical significance (p < 0.05). The table was generated by the authors using Stata version 18 [22]

Variable	Adjusted Odds Ratio	95% CI	P-value
Prescription pain medication use (yes versus no)	1.54	1.15-2.07	0.005*
Age (years)	1.02	1.01-1.03	0.003*
Sex (female versus male)	0.91	0.63-1.31	0.587
Income-to-poverty ratio	0.92	0.85-0.99	0.029*
Health insurance (yes versus no)	1.26	0.82-1.95	0.279
Physical activity (active versus not active)	0.53	0.39-0.74	<0.001*
Education level	-	-	-
Less than high school versus college or above	1.42	0.95-2.13	0.085
High school/GED versus college or above	1.63	1.11-2.39	0.015*
Race/ethnicity	-	-	-
Mexican American versus non-Hispanic White	0.74	0.50-1.10	0.129
Other Hispanic versus non-Hispanic White	0.69	0.40-1.20	0.176
Non-Hispanic Black versus non-Hispanic White	1.16	0.87-1.55	0.308
Non-Hispanic Asian versus non-Hispanic White	0.29	0.18-0.48	<0.001*
Other race versus non-Hispanic White	1.75	0.84-3.61	0.126

The results indicate that prescription pain medication use was associated with higher odds of high cardiovascular risk, with an adjusted odds ratio of 1.54 (95% CI, 1.15-2.07; p = 0.005). Increasing age was also associated with higher odds, with an odds ratio of 1.02 (95% CI, 1.01-1.03; p = 0.003). A higher income-to-poverty ratio was associated with lower odds of high cardiovascular risk, with an odds ratio of 0.92 (95% CI, 0.85-0.99; p = 0.029). Physical activity showed a protective association, with active individuals having lower odds of high cardiovascular risk, with an odds ratio of 0.53 (95% CI, 0.39-0.74; p < 0.001).

Sex and health insurance status were not significantly associated with the outcome. In terms of education, individuals with a high school education had higher odds of high cardiovascular risk compared to those with a college education, with an odds ratio of 1.63 (95% CI, 1.11-2.39; p = 0.015), while those with less than a high school education did not show a statistically significant difference.

For race and ethnicity, non-Hispanic Asian participants had lower odds of high cardiovascular risk compared to non-Hispanic White participants, with an odds ratio of 0.29 (95% CI, 0.18-0.48; p < 0.001). Other racial and ethnic groups did not show statistically significant differences.

**Figure 1:**
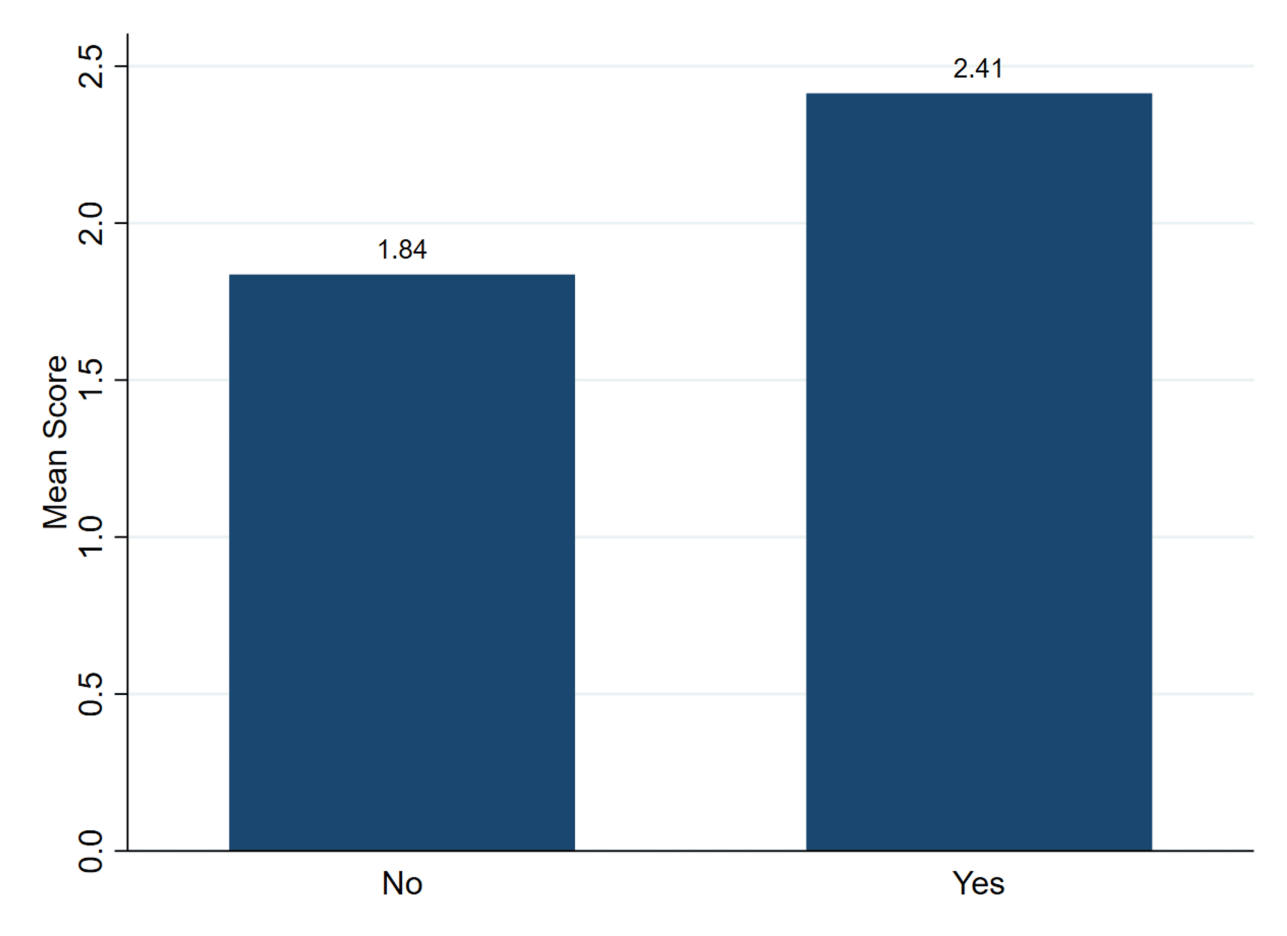
Mean cardiovascular risk score by prescription pain medication use Values represent survey-weighted mean cardiovascular risk scores. The composite score ranges from 0 to 5 and reflects the number of cardiovascular risk factors present, including hypertension, diabetes, dyslipidemia, obesity, and smoking. Analyses account for sampling weights, strata, and clusters

Figure 1 illustrates the distribution of the mean cardiovascular risk score according to prescription pain medication use.

The participants who reported prescription pain medication use had a higher mean cardiovascular risk score (2.41) compared to non-users (1.84). This indicates a greater clustering of cardiovascular risk factors among medication users, supporting the study finding that this group carries a higher overall cardiometabolic risk burden.

## Discussion

In this study, prescription pain medication use was associated with higher odds of high cardiovascular risk among US adults. Individuals reporting pain medication use were older, had lower income levels, and showed a higher prevalence of major cardiovascular risk factors, including obesity, diabetes, and hypertension. The distribution of the composite cardiovascular risk score also showed a greater proportion of individuals in the high-risk category among medication users. These findings align with existing evidence indicating that cardiovascular risk factors remain highly prevalent in the United States and often cluster within vulnerable populations [1,2]. The higher burden of obesity and metabolic conditions observed among medication users is consistent with prior studies showing strong links between obesity, chronic disease, and cardiovascular risk [3]. The observed differences in hypertension and diabetes prevalence are also clinically relevant, given their established role in increasing cardiovascular morbidity and mortality [4,5]. The clustering of cardiometabolic risk factors among pain medication users has important implications for routine clinical care, particularly as chronic pain populations often present with overlapping conditions such as obesity, hypertension, dyslipidemia, and impaired glucose metabolism. National data demonstrate that cardiometabolic risk factors frequently coexist and remain suboptimally controlled among US adults, underscoring the need for more comprehensive and proactive management strategies [1,2]. In patients with chronic pain, this burden is further amplified, as pain-related functional limitations, medication use, and associated behavioral factors contribute to higher rates of cardiovascular risk factor clustering [3,13,15]. These findings suggest that patients presenting for pain management, whether in primary care or specialty settings, should not be evaluated solely for pain symptoms but rather through a broader cardiometabolic lens. 

The association between prescription pain medication use and cardiovascular risk may reflect underlying patterns of chronic pain and comorbidity rather than a direct effect of the medications themselves. Prior work has shown that prescription medication use is common among individuals with chronic pain conditions, who often have multiple coexisting health conditions [6,15]. Chronic pain has been linked to higher body mass index, reduced physical activity, and adverse health behaviors, all of which contribute to cardiovascular risk [13,14]. The higher rates of smoking and physical inactivity observed in this study among medication users further support this pattern. Chronic pain plays a significant role in shaping patient behavior, functional capacity, and overall health, which may contribute to the accumulation of cardiovascular risk factors. People with persistent pain often experience reduced mobility, physical limitations, and fatigue, which can significantly impair their ability to engage in regular physical activity [13]. This sedentary behavior is a well-established contributor to obesity, insulin resistance, and overall cardiovascular risk. In this study, the noticeably higher prevalence of physical inactivity among individuals using prescription pain medication reflects this functional limitation and shows a pathway through which cardiovascular risk rises.

In addition to physical activity, chronic pain is associated with behavioral and psychological factors that influence cardiovascular health. Persistent pain may increase the risk of adverse health behaviors such as smoking and poor dietary habits, as well as reduced adherence to health-promoting activities, and other such maladaptive coping behaviors [14]. These behavioral patterns are shown in the study, where higher rates of smoking and lower levels of physical activity were observed among individuals using pain medications.

Additionally, emerging evidence suggests that opioid use may be associated with adverse cardiovascular outcomes and metabolic changes, although the mechanisms remain under investigation [17,18]. Long-term opioid exposure has also been linked to endocrine changes that may influence metabolic regulation and cardiovascular risk profiles [12]. For non-opioid analgesics, particularly nonsteroidal anti-inflammatory drugs, there is established evidence of increased cardiovascular risk, including elevated blood pressure and the increased risk of cardiovascular events [10,11]. These pathways provide a plausible explanation for the higher clustering of cardiovascular risk factors observed among individuals using prescription pain medications.

Current US guidelines emphasize the importance of identifying and managing cardiovascular risk factors in adults, particularly those with multiple comorbid conditions. The American Heart Association framework for cardiovascular health highlights the role of modifiable risk factors, including smoking, obesity, physical inactivity, and metabolic conditions, in determining cardiovascular risk [1]. These recommendations support a comprehensive risk assessment rather than focusing on single conditions. In the context of this study, the higher burden of cardiovascular risk factors among individuals using prescription pain medications suggests that this population may benefit from closer risk factor monitoring and integrated care approaches. The findings are also consistent with national trends showing persistent challenges in achieving optimal cardiovascular health across the adult population [2].

Several biological and behavioral pathways may explain the observed association between prescription pain medication use and cardiovascular risk. Chronic pain can lead to reduced mobility and lower levels of physical activity, which in turn contribute to weight gain and metabolic dysfunction [13]. Psychological stress and comorbid mental health conditions associated with chronic pain may also influence smoking behavior and other lifestyle factors [14]. Opioids may affect hormonal regulation, including the suppression of endocrine function, which can alter metabolism and cardiovascular risk profiles [12]. In addition, some analgesic medications may have direct effects on blood pressure, lipid metabolism, or vascular function [10,11]. These factors likely interact and contribute to the clustering of cardiovascular risk factors observed in this population, though causality cannot be established within a cross-sectional design. These findings reflect associations observed in cross-sectional data and should not be interpreted as evidence of causality.

Strengths and limitations of the study

This study has several strengths, including the use of nationally representative data and standardized measures from NHANES, which enhance generalizability. The analysis accounted for complex survey design and used a composite cardiovascular risk measure that reflects multiple clinically relevant factors.

However, important limitations should be considered. The cross-sectional design does not allow for the temporal assessment of exposure and outcome. Several variables, including medication use, smoking status, and physical activity, were based on self-report and may be subject to misclassification. Missing data were addressed using a complete case approach, which substantially reduced the analytic sample. This may introduce selection bias if the participants with complete data differ systematically from those with missing information. For example, individuals with poorer health status or lower socioeconomic position may be more likely to have incomplete data, potentially leading to the underestimation or overestimation of the association. As a result, the findings should be interpreted with caution, as the analytic sample may not fully represent the target population despite the use of survey weights.

Residual confounding is also a key consideration. Although the analysis was adjusted for several demographic and socioeconomic factors, unmeasured or incompletely measured variables may influence both prescription pain medication use and cardiovascular risk. These include factors such as pain severity, duration of and indication for medication use, comorbid conditions, healthcare utilization patterns, prescribing practices, and underlying disease burden. Behavioral and lifestyle factors, including diet quality, physical activity intensity, and medication adherence, were also not fully captured. As a result, confounding by indication may be present, where individuals receiving pain medications differ systematically from non-users in ways that are related to cardiovascular risk. These limitations may affect the precision of the observed associations.

Future research should consider longitudinal designs and incorporate more detailed measures of medication exposure, clinical indications, and behavioral risk factors to better understand these relationships and reduce the impact of residual confounding.

## Conclusions

This study highlights that prescription pain medication use is associated with a higher burden of cardiovascular risk factors among US adults. Individuals reporting medication use showed the greater clustering of modifiable risk factors, including metabolic and behavioral conditions that are central to cardiovascular health. These findings support the need for careful cardiovascular risk assessment in patients receiving prescription pain medications, particularly in those with multiple comorbid conditions. Integrating risk factor screening into routine care for individuals with chronic pain may improve the early identification of elevated risk. Future research should use longitudinal designs to clarify temporal relationships and incorporate detailed measures of medication exposure, lifestyle behaviors, and pain characteristics to better understand these associations.
